# Supplementary material for: Electric field–induced pore constriction in the human Kv2.1 channel
Source: Proc Natl Acad Sci U S A. 2025 May 14;122(20):e2426744122. doi: 10.1073/pnas.2426744122 (PMC12107148; doi:10.1073/pnas.2426744122)
Supplement: Supplementary file 1 — Appendix 01 (PDF) [file pnas.2426744122.sapp.pdf]

Supporting Information for

Electric field-induced pore constriction in the human K_v2.1 channel.

Venkata Shiva Mandala[1] and Roderick MacKinnon[1, *].

[1]Laboratory of Molecular Neurobiology and Biophysics, Howard Hughes Medical Institute, The Rockefeller University, New York, United States.

*Correspondence to: Roderick MacKinnon.

Email: mackinn@rockefeller.edu.

This PDF file includes:

Figures S1 to S5
Table S1
Legends for Movies S1 to S2

Other supporting materials for this manuscript include the following:

Movie S1
Movie S2

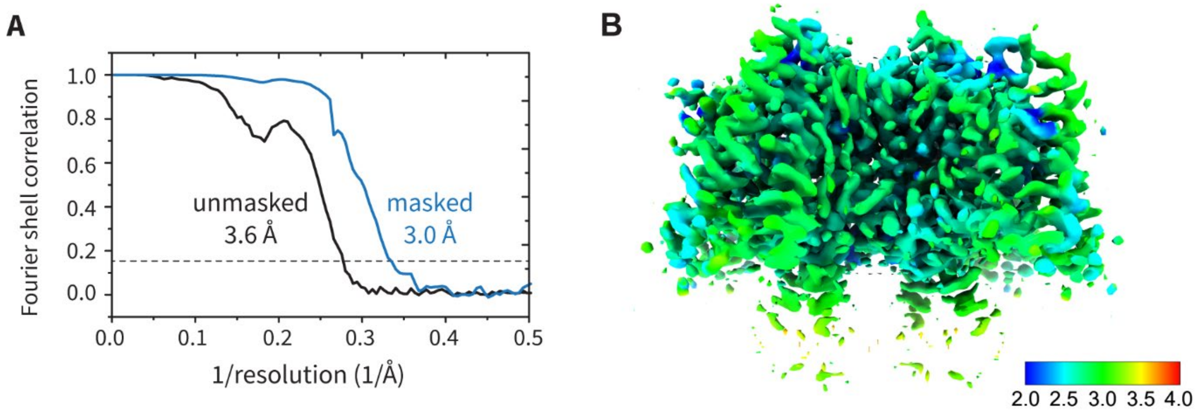

Figure S1. Fourier shell correlation curves and local resolution estimates for the depolarized map from the unpolarized dataset (i.e. the depolarized-highK conformation with all four voltage sensors up and an open pore).

(**A**) Fourier Shell Correlation (FSC) curves for the depolarized map from the unpolarized dataset calculated using the two independent half-maps from refinement. The FSC curve for the masked map is shown in *blue* and that for the unmasked map is in *black*. The nominal resolution at the gold-standard criterion (FSC=0.143, dashed *black* line) is indicated. (**B**) CryoSPARC-derived local resolution estimates (FSC = 0.143) for the same map overlaid with the sharpened map. The color key indicating the local resolution estimates is given in the bottom right corner.

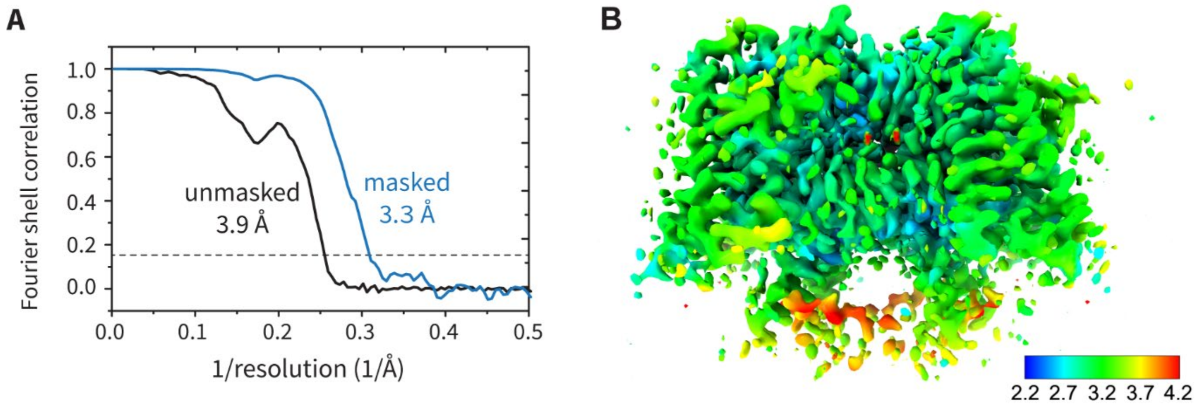

Figure S2. Fourier shell correlation curves and local resolution estimates for the depolarized map from the polarized dataset (i.e. the depolarized-lowK conformation with all four voltage sensors up and an open pore).

(**A**) Fourier Shell Correlation (FSC) curves for the depolarized map from the polarized dataset calculated using the two independent half-maps from refinement. The FSC curve for the masked map is shown in *blue* and that for the unmasked map is in *black*. The nominal resolution at the gold-standard criterion (FSC=0.143, dashed *black* line) is indicated. (**B**) CryoSPARC-derived local resolution estimates (FSC = 0.143) for the same map overlaid with the sharpened map. The color key indicating the local resolution estimates is given in the bottom right corner.

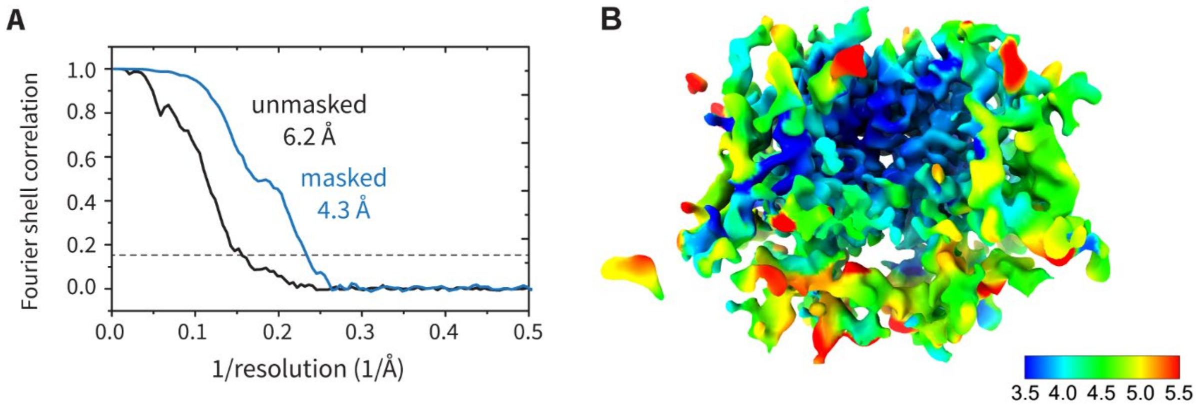

Figure S3. Fourier shell correlation curves and local resolution estimates for the hyperpolarized intermediate map from the polarized dataset (i.e. the conformation with 2 voltage sensors down and an open pore).

(**A**) Fourier Shell Correlation (FSC) curves for the hyperpolarized intermediate map from the polarized dataset calculated using the two independent half-maps from refinement. The FSC curve for the masked map is shown in *blue* and that for the unmasked map is in *black*. The nominal resolution at the gold-standard criterion (FSC=0.143, dashed *black* line) is indicated. (**B**) CryoSPARC-derived local resolution estimates (FSC = 0.143) for the same map overlaid with the sharpened map. The color key indicating the local resolution estimates is given in the bottom right corner.

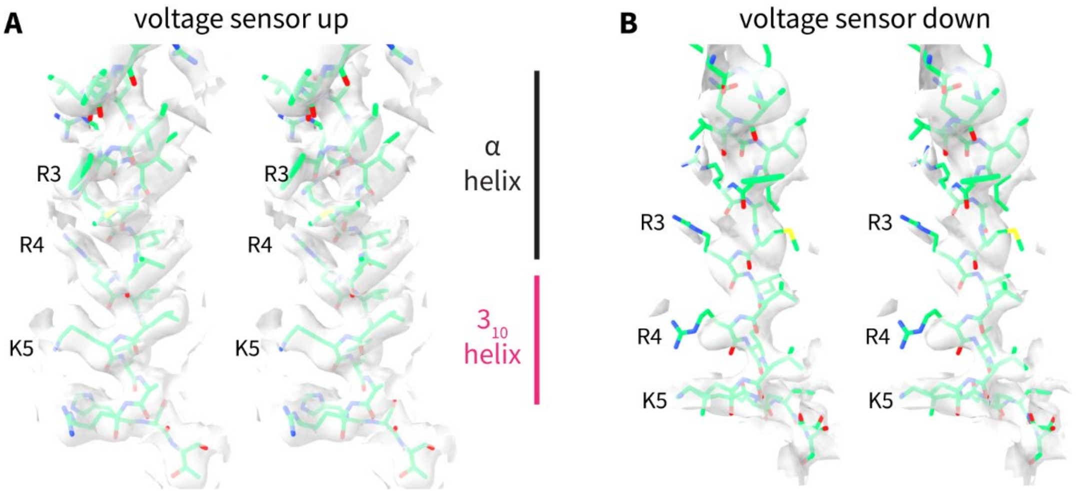

Figure S4. Comparison of the S4 helix in the voltage sensor in the up and down conformations.

(**A, B**) Stereoviews showing the S4 helix and associated cryo-EM density maps of the (**A**) depolarized conformation and the (**B**) hyperpolarized intermediate conformation, both from the polarized dataset. The helix is shown in *green* stick representation and the density map is shown as a translucent *grey* surface. The locations of three positive charges (R3, R4, and K5), and the α-helical and 3_{10}-helical segments are indicated.

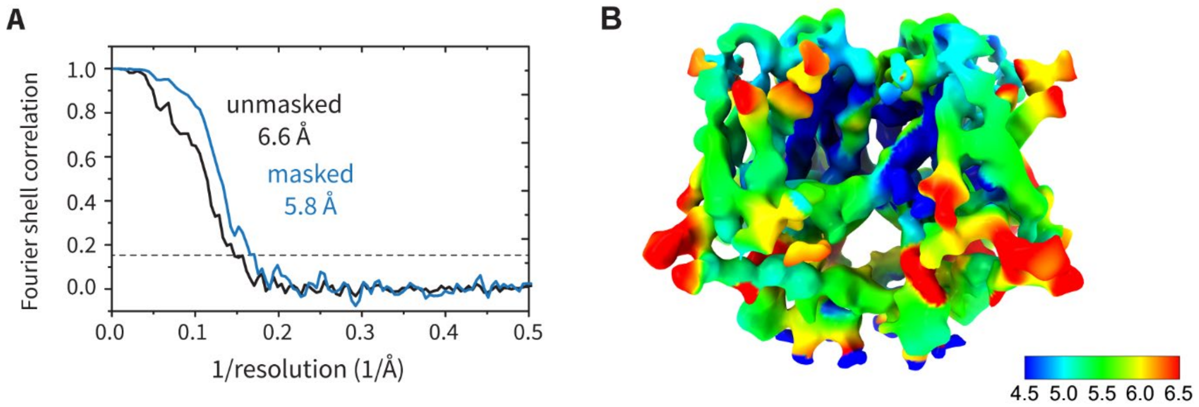

Figure S5. Fourier shell correlation curves and local resolution estimates for the hyperpolarized constricted map from the polarized dataset (i.e. the conformation with 4 voltage sensors down and a constricted pore).

(**A**) Fourier Shell Correlation (FSC) curves for the hyperpolarized constricted map from the polarized dataset calculated using the two independent half-maps from refinement. The FSC curve for the masked map is shown in *blue* and that for the unmasked map is in *black*. The nominal resolution at the gold-standard criterion (FSC=0.143, dashed *black* line) is indicated. (**B**) CryoSPARC-derived local resolution estimates (FSC = 0.143) for the same map overlaid with the sharpened map. The color key indicating the local resolution estimates is given in the bottom right corner.

Table S1. Summary of cryo-EM reconstruction and structural model statistics.

Reconstruction	Depolarized_highK	Depolarized_lowK	Intermediate	Constricted
Dataset	Unpolarized	Polarized		
Microscope/Camera	Titan Krios 2 300 kV, Gatan K3	Titan Krios 3 300 kV, Falcon IVi		
Pixel Size	0.844 Å	0.743 Å		
Total dose	60 $e^-/Å^2$	60 $e^-/Å^2$		
Defocus range	-1.0 to -2.0 µm	-0.8 to -1.8 µm		
Movies collected	17,007	20,339		
Particle number	65,670	47,718	13,810	2,251
Symmetry imposed	C4	C4	C1	C4
Nominal resolution (masked)	3.0 Å	3.3 Å	4.3 Å	5.8 Å
**Models**	**Depolarized_highK**	**Depolarized_lowK**	**Intermediate**	**Constricted**
**Ramachandran**				
Preferred (%)	98.47	97.97	95.66	96.91
Allowed (%)	1.53	2.03	4.34	3.09
Outliers (%)	0.00	0.00	0.00	0.00
**MolProbity**				
Clash Score	4.31	3.15	4.20	4.90
Rotamer Outliers (%)	0.23	0.11	1.19	0.00
Cβ deviations	0.00	0.00	0.00	0.00
Overall Score	1.21	1.11	1.56	1.44
**RMS deviations**				
Bond lengths (Å)	0.003	0.004	0.003	0.003
Bond angles (°)	0.46	0.49	0.47	0.50

Movie S1 (separate file). Sequence of conformational changes occurring during membrane hyperpolarization.

The movie shows a side view of the morph between two structures of K_v2.1: with the pore open and voltage sensors up (the depolarized-lowK conformation), and with the pore constricted and voltage sensors down (the hyperpolarized constricted conformation). The protein is shown in Cα trace representation. The S4 and S6 of one subunit are colored cyan and green, respectively. The Cα positions of positive-charged residues in the S4 helix are shown as blue spheres.

Movie S2 (separate file). Sequence of conformational changes occurring during membrane hyperpolarization.

The movie shows a top-down view (from the extracellular side) of the morph between two structures of K_v2.1: with the pore open and voltage sensors up (the depolarized-lowK conformation), and with the pore constricted and voltage sensors down (the hyperpolarized constricted conformation). The protein is shown in Cα trace representation. The S4 and S6 of one subunit are colored cyan and green, respectively. The Cα positions of positive-charged residues in the S4 helix are shown as blue spheres. The Cβ of P410 – the narrowest rigid constriction in the channel axis – is shown as orange spheres.
